# A hybrid MBE-based growth method for large-area synthesis of stacked hexagonal boron nitride/graphene heterostructures

**DOI:** 10.1038/srep43644

**Published:** 2017-02-27

**Authors:** Joseph M. Wofford, Siamak Nakhaie, Thilo Krause, Xianjie Liu, Manfred Ramsteiner, Michael Hanke, Henning Riechert, J. Marcelo J. Lopes

**Affiliations:** 1 Paul-Drude-Institut für Festkörperelektronik, Hausvogteiplatz 5–7, 10117 Berlin, Germany; 2 Department of Physics, Chemistry and Biology, Linköping University, SE-58183 Linköping, Sweden

## Abstract

Van der Waals heterostructures combining hexagonal boron nitride (h-BN) and graphene offer many potential advantages, but remain difficult to produce as continuous films over large areas. In particular, the growth of h-BN on graphene has proven to be challenging due to the inertness of the graphene surface. Here we exploit a scalable molecular beam epitaxy based method to allow both the h-BN and graphene to form in a stacked heterostructure in the favorable growth environment provided by a Ni(111) substrate. This involves first saturating a Ni film on MgO(111) with C, growing h-BN on the exposed metal surface, and precipitating the C back to the h-BN/Ni interface to form graphene. The resulting laterally continuous heterostructure is composed of a top layer of few-layer thick h-BN on an intermediate few-layer thick graphene, lying on top of Ni/MgO(111). Examinations by synchrotron-based grazing incidence diffraction, X-ray photoemission spectroscopy, and UV-Raman spectroscopy reveal that while the h-BN is relaxed, the lattice constant of graphene is significantly reduced, likely due to nitrogen doping. These results illustrate a different pathway for the production of h-BN/graphene heterostructures, and open a new perspective for the large-area preparation of heterosystems combining graphene and other 2D or 3D materials.

Devices based on the graphene/hexagonal boron nitride (h-BN) materials system offer a host of potential advantages, including high speeds, extremely low power consumption, and various novel functionalities^1^. Much of this promise arises from the intrinsic properties of graphene, although h-BN plays a crucial role in facilitating their effective utilization. Whereas many conventional dielectrics, such as SiO_2_, partially mask the useful properties of graphene, the atomically smooth surface and homogeneous charge potential offered by h-BN allow their fullest expression^2^,^3^. Hexagonal boron nitride can also serve purposes beyond that of simple passive mechanical support – such as a tunneling barrier^4^,^5^ – enabling an even wider variety of device architectures. These considerations have drawn the focus of researchers towards the exploration and effective production of heterostructures containing graphene and h-BN.

Graphene and h-BN share the same layered crystal structure and hexagonal in-plane symmetry, with a lattice mismatch of only ~1.7%. Despite this inherent structural compatibility, most successful demonstration heterostructures have relied on stacked flakes which have been mechanically exfoliated from bulk samples of the two materials^6^,^7^,^8^,^9^ However, this method is inherently unscalable, and any interface contamination introduced during the transfer process would adversely impact the properties of final devices. Therefore, the direct synthesis of large-area graphene and h-BN through additive growth will ultimately be required. Many attempts aiming at the preparation of vertical heterostructures combining graphene and h-BN by employing different methods have been reported^10^,^11^,^12^,^13^,^14^,^15^,^16^,^17^,^18^,^19^,^20^. Despite the considerable progress that has been achieved, the realization of heterostructure films (graphene on h-BN or h-BN on graphene) which offer high crystalline quality and are continuous over large areas remains a central challenge. In particular, these results suggest that the synthesis of continuous h-BN on top of graphene poses additional difficulties when compared to growing graphene on h-BN^17^,^18^. Unlike heterostructure growth, the literature contains many instances of graphene and h-BN being synthesized alone using both chemical and physical vapor deposition methods (CVD and PVD, respectively); experiments which have made clear that metal substrates offer the chemical environment most conducive to the synthesis of high-quality films of both materials. Examples include the widely replicated CVD growth of graphene on Cu foil substrates (among many others)^21^, as well as h-BN synthesis on Ni^22^, Pt(111)^23^, Cu^24^, and more. Metallic substrates can support the growth of fully epitaxial films of both graphene^25^ and h-BN^23^, and individual crystalline domains millimeters across even in the absence of epitaxy^26^. This is in contrast to graphene and h-BN films deposited on dielectric substrates, which typically results in limited domain sizes and substantial crystalline disorder^27^,^28^. The efficacy of metallic substrates for the production of high-quality films of graphene and h-BN points to a problem inherent to the production of multilayer heterostructures: once the substrate is covered by the first material, the favorable environment it provides is no longer accessible for the growth of the subsequent material.

In this manuscript we demonstrate a method for the production of h-BN/graphene heterostructures which allows both materials to form on the surface of the Ni substrate. Recently we have shown that Ni is an effective substrate for the growth of both atomically thin h-BN and graphene via molecular beam epitaxy^29^,^30^. Here we exploit the finite solubility of C in Ni^31^ by first saturating a Ni film (grown on MgO(111)), then depositing a few-monolayer thick h-BN film from elemental B and N on the exposed Ni surface, and finally ramping the sample temperature down to controllably precipitate the C and form few-layer graphene at the interface between the h-BN and Ni. The resulting heterostructure film is composed of a top layer of h-BN on an intermediate layer of graphene, supported by the Ni/MgO(111) substrate. This method may be generalized to achieve the controlled, high-quality formation of various heterosystems combining graphene with other materials, especially in instances where the direct 2D or 3D film growth on top of a graphene-covered surface might not be feasible.

## Results and Discussion

**Figure 1:**
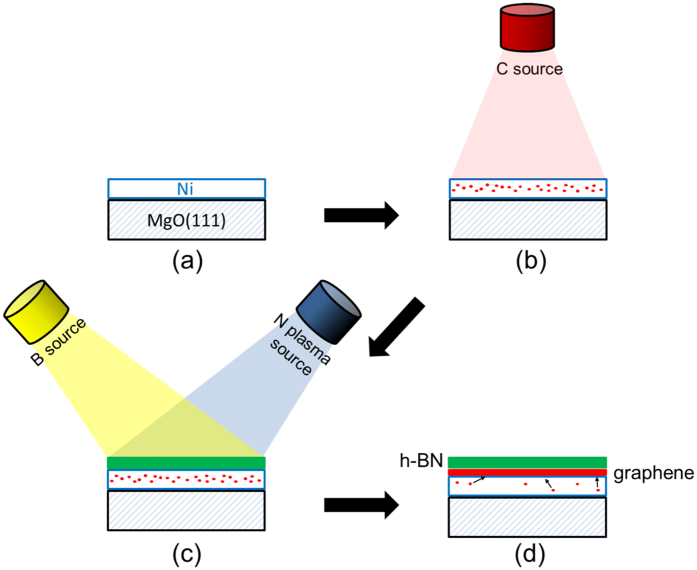
Schematic illustration of the synthesis process utilized here for h-BN/graphene heterostructures on Ni/MgO(111) templates. (**a**) Starting with a blank Ni/MgO(111) substrate, (**b**) C is dissolved into the Ni film by e-gun evaporation at high temperature. (**c**) This is followed by the MBE growth of a few-layer thick h-BN film on the exposed Ni surface utilizing N-plasma and elemental B. (**d**) The last step consists of forcing C precipitation from the Ni film by controlled sample cooling, resulting in few-layer graphene forming as a continuous film at the h-BN/Ni(111) interface. Note that the illustrations are not to scale. For the sake of simplicity, possible precipitation of C at the Ni/MgO interface is not considered in the illustrations.

The main steps involved in the fabrication of the h-BN/graphene heterostacks studied here are illustrated schematically in Fig. 1. Substrates were prepared by depositing at room temperature 300 nm of Ni on 1 cm^2^ pieces of MgO(111) by electron beam evaporation, and back-coating them with 1 μm of Ti. The Ni/MgO(111) templates were transferred through air to a UHV MBE system (~1 × 10^−10^ Torr) where they were outgassed at 300 °C for 60 minutes, and sputtered with Ar (1 × 10^−4^ Torr, 2 kV, 10 mA emission current). The films were then annealed at 850 °C for 20 minutes and cooled to 730 °C for C saturation, which was accomplished by evaporating elemental C from an electron beam heated graphite target. The amount of carbon utilized for the saturation step was chosen to avoid the concomitant formation of graphene at the Ni surface^32^,^33^, with the intent of allowing the subsequent h-BN growth to occur directly on the bare Ni surface. Hexagonal boron nitride was then grown using elemental B and N from a high-temperature effusion cell and an RF plasma source, respectively. Additional details about the process can be found elsewhere^30^. Deposition was halted once the h-BN film reached an average thickness of ~3 monolayers (ML, as calibrated by X-ray reflectivity and atomic force microscopy (AFM) profilometry). Finally, few-layer thick graphene (2 to 3 ML) was formed at the interface between the h-BN and Ni from the C previously dissolved in the Ni film by cooling the sample at 4 °C per minute.

**Figure 2:**
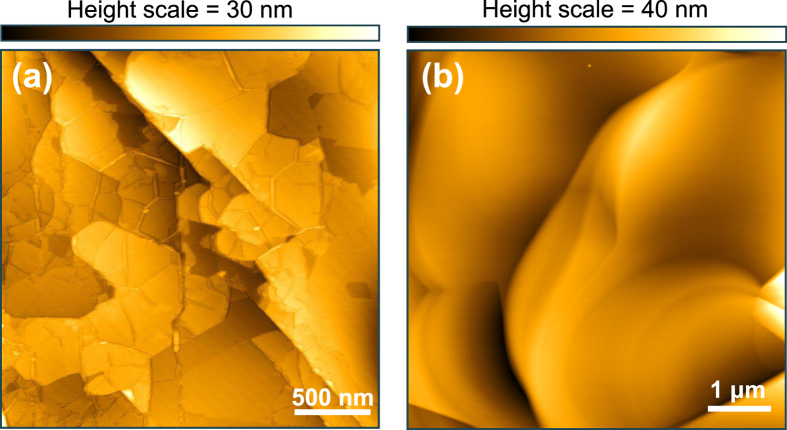
(**a**) AFM image of a h-BN/graphene heterostructure film grown on Ni/MgO(111). The surface shows the typical features resulting from the growth of a 2D material on a metal template (wrinkles), as well as topographic variations such as step edges which originate from the surface of the underlying Ni film. (**b**) AFM image of a bare Ni/MgO(111) template after annealing at 850 °C for 20 minutes (without subsequent growth). The surface contains typical topographic features of the Ni film surface such as step edges and flat terraces. However, no wrinkles are observed.

AFM scans of the h-BN/graphene heterostructure samples show the network of wrinkles typically seen in two-dimensional films grown on metallic substrates (see an example in Fig. 2), which is very similar to what we have previously observed for continuous graphene and h-BN prepared by MBE on Ni^29^,^30^. The formation of wrinkles takes place during cooling and is mainly related to the unequal expansion coefficients of the 2D materials and Ni. Hence, the wrinkle structure, which is observed in all AFM measurements performed at different locations of the sample surface, indicates that a continuous film is formed after employing the synthesis method described in Fig. 1. Note that over larger scales the surface topology is dominated by the features of the underlying Ni film, including step clusters and grain boundaries. Height variations seen in the AFM image are mainly related to these, as well as to localized thickness inhomogeneities in the h-BN and graphene coverage. Fig. 2b shows, for comparison, an AFM height image of a bare Ni/MgO(111) prepared in the same manner but without subsequent heterostructure growth.

**Figure 3:**
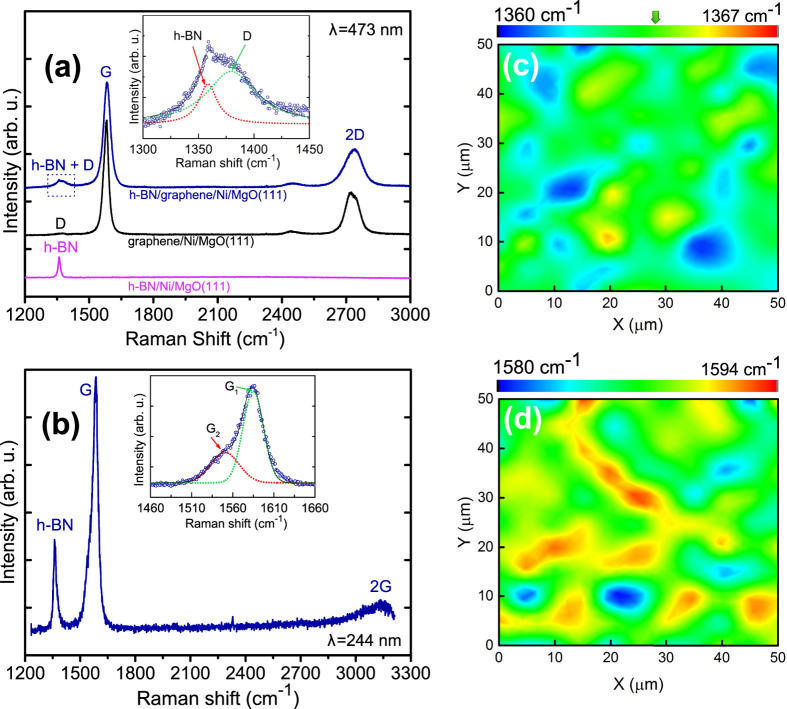
(**a**) Raman spectra of a h-BN/graphene heterostructure film, as well as of pure graphene and h-BN films also grown by MBE. All spectra were excited at λ = 473 nm. Note that for the h-BN spectrum the Ni-related background was subtracted. The inset shows a magnification for the heterostructure film spectrum of the region where the D peak of graphene and h-BN related peak overlap. The contribution located at ~2450 cm^−1^ is the D + D” peak related to graphene which is not discussed in this work (detailed information about this mode can be found elsewhere^79^). (**b**) UV Raman spectrum of heterostructure excited at λ = 244 nm. The inset shows a magnification of the region around the G peak. The G peak is fitted with two Gauss functions (G_1_: green dashed line and G_2_: red dashed line). (**c**) and (**d**) Mappings of the position of the h-BN phonon line and of the G_1_ peak of graphene, respectively. They were both acquired for the same 50 μm × 50 μm area. The arrow in (**c**) shows the peak position of bulk h-BN at 1364 cm^−1^ according to literature. The corresponding UV Raman mapping were recorded with excitation at λ = 244 nm. The signal to noise ratio for the mappings in (**c**) and (**d**) was always larger than 100.

Figure 3a presents a Raman spectrum from a h-BN/graphene heterostructure showing the scattering peaks characteristic of both materials. The G and 2D peaks of the few-layer graphene spectrum are clearly defined, and the defect-related D peak has a low relative intensity. The D peak is probably associated to the defects originated from an unintentional N-doping of the underlying graphene, as it will be discussed later. For comparison, the Raman spectra obtained from pure MBE grown graphene as well as pure MBE grown h-BN, using the same growth parameters as for the heterostructure constituents are also presented in Fig. 3a. (see section Methods for details). For the heterostructure case, it is immediately apparent that the Raman signal originating from the graphene layer is significantly more intense than that from the h-BN film. This is to be expected because even though the two materials are present in approximately the same quantity, scattering from the graphene film is resonant with excitation lasers in the optical range (λ = 473 nm here, or E = 2.62 eV), while the h-BN is not. The observation of the h-BN signal in the combined heterostructure spectrum is further complicated by the overlap of its characteristic peak with the D peak of graphene. However, a closer examination of the relevant spectral range (inset in Fig. 3a) clearly shows the Raman intensity to be composed of separate and distinct peaks, confirming the presence of both materials. The narrow FWHM of the h-BN peak, ~12 cm^−1^, is also consistent with the high crystalline quality of the h-BN^34^. When using the 473 nm laser the Raman signal from graphene was observed regardless of where the spectrum was collected, while the h-BN Raman peak was possible to resolve only at ~20% of the locations examined, possibly coinciding with locally thicker regions in the h-BN film. Raman mapping using this laser line was also not possible due to the presence of spatially inhomogeneous luminescence coming from the Ni substrate^30^.

Unlike Raman scattering in the visible spectrum, UV excitation (λ = 244 nm, or E = 5.08 eV) allowed the continuity of the h-BN over the full heterostructure to be verified. Figure 3b depicts a typical Raman spectrum from a h-BN/graphene film excited with the UV laser. The signal related to the E_2g_ optical phonon of h-BN is enhanced, since the efficiency of Raman scattering from h-BN exhibits a certain degree of resonance for excitation at 5.08 eV^35^. In addition, previous studies have shown that both the D and the 2D peak of graphene exhibit a linear blue-shift as well as a strong decrease in intensity with increasing excitation energy, while the G peak position remains unaltered^36^,^37^,^38^,^39^. Considering the linear dispersion suggested in literature^36^, the D peak of graphene is expected at ~1485 cm^−1^ when the 5.08 eV laser is used. Thus, the well-defined peak observed at ~1364 cm^−1^ originates purely from the h-BN in the heterostructures. This peak was always detected in numerous UV Raman point measurements and mappings regardless of the position being measured, which indicates that the h-BN forms as a continuous layer rather than as isolated islands. A representative map of the position of this peak over a 50 μm × 50 μm area is shown in Fig. 3c. The h-BN’s Raman signal is always clearly observed in the presented map with a signal to noise ratio typically around 500 and always better than 100. The frequency of the h-BN phonon line is found to be centered at ~1364 cm^−1^ (consistent with the reported values for bulk h-BN^34^,^35^) with a total variation between 1360 and 1367 cm^−1^. We did not observe any significant intensity near 1485 cm^−1^ in Raman spectra collected from the heterostructures; consistent with the quenching of the graphene’s D peak in UV Raman measurements^36^. The broad band observed at ~3150 cm^−1^ (labeled as 2 G) is attributed to a non-resonant second-order Raman scattering in graphene^40^. The 2D peak could not be detected in the UV Raman measurements, which is also consistent with previous studies^38^,^40^,^41^. The most prominent peak present in all UV Raman spectra is the G peak of graphene. Interestingly, it has an asymmetric shape and is composed of two components: a more prominent component observed within the 1580–1594 cm^−1^ range (G_1_), and a small shoulder centered at 1555 ± 20 cm^−1^ (G_2_ - see the following discussion for further details). Figure 3d shows a mapping of the position of the G_1_ component, which was taken at the exact same position as the h-BN map (Fig. 3c). As in the case of h-BN, the uninterrupted and clear detection of this graphene-related peak in numerous measurements performed at different surface positions serves to demonstrate the lateral continuity of the graphene formed underneath the h-BN. Raman measurements performed in h-BN/graphene heterostructure films which were transferred onto SiO_2_/Si substrates yielded very similar results (see Figures S1 and S2 in Supplementary Information). In that case, it was also possible to perform Raman mappings using visible light excitation as a Ni-related background signal was not present. Finally, the Raman results obtained from the heterostructures serve as a evidence for the formation of few-layer graphene at h-BN/Ni interface and not on top of the h-BN. Previous studies have shown that at similar growth temperatures, even direct deposition of C on top of exfoliated h-BN single crystals result in highly defective and localized graphene (i.e. exhibiting a D peak that is more intense than the G peak)^16^,^42^, which is not the case for the heterostructures reported here. The stacking order of graphene and h-BN layers was additionally verified by exposing a heterostructure sample to O_2_-Plasma treatment which etches graphene and preserves h-BN. Raman measurements show no change in the graphene signal for surface areas exposed to O_2_-plasma etching (see Supplementary Information Fig. S3).

As already pointed out, our Raman investigations provide evidence for the presence of unintentional modifications in the few-layer graphene film formed at the interface. In fact, for our growth experiments, graphene doping and/or alloying close to the h-BN/graphene interface cannot be ruled out. During the growth of h-BN (see Fig. 1c), when B and N are exposed to the hot surface of Ni, it is possible that undissolved C atoms and even a few graphene inclusions from the previous saturation step (see Fig. 1b) are present at the Ni surface. In this environment, a stronger interaction between C and N is anticipated considering a relatively strong reactivity of N species generated by the plasma source in comparison to elemental B. In this case, the main modification taking place in the few-layer graphene would be doping with N which is probably inhomogeneous laterally and along the growth direction. In addition to the existence of a low-intensity D peak, which is consistent with the existence of defects originated from N-doping^43^, the observed position of the G_1_ peak (see Fig. 3d) is generally blue-shifted as compared to the one of pristine graphene (at ~1580 cm^−1^)^44^, which suggests that N and/or B atoms have been incorporated into the graphene film as dopants^43^,^45^,^46^. Furthermore, the splitting of the G peak into two components indicates the existence of a doping (charge density) gradient across the few-layer graphene film, which induces a dipole formation between the topmost and bottom graphene layers. The inversion symmetry breaking induced by such gradients is known to result in a G peak splitting^47^,^48^. An alternative explanation for the low-frequency G_2_ peak is a possible alloying of the h-BN and graphene films close to their interface. In previous work, the observation of a red shift and broadening of the G peak in h-BNC alloys with respect to pure graphene has been reported^49^,^50^. Uddin *et al*. recently proposed that the G peak of graphene downshifts (towards the h-BN phonon line), when a homogeneous single phase alloy of h-BNC is formed^51^. Nevertheless, the overall shape of the Raman spectra (e.g. broad D and G peaks and high D peak intensity) reported by these studies and more recently by Meng *et al*.^52^, also for h-BNC alloy films, is different from what is observed in the present case. This and the X-ray photoemission spectroscopy (XPS) and grazing incidence X-ray diffraction (GID) results shown next, indicates that alloying does not take place in the heterostructure films studied here.

**Figure 4:**
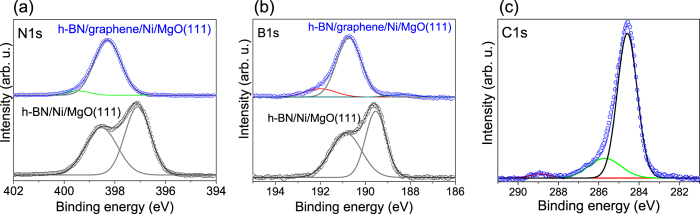
XPS core level spectra of the (**a**) N1s, (**b**) B1s, and (**c**) C1s regions for h-BN (**a**,**b**) and h-BN/graphene heterostructure films (**a–c**) synthesized on Ni/MgO(111) templates.

XPS analyses provided information on the chemical composition and bonding nature of the MBE-grown material. Figure 4 displays the N1s, B1s, and the C1s spectral regions obtained from the h-BN/graphene heterosystem. For comparison, the N1s and B1s regions of a h-BN film grown on a Ni/MgO(111) template utilizing the exact same parameters (but without the C saturation step illustrated in Fig. 1b) is also presented. For the latter, both the N1s (Fig. 4a) and B1s (Fig. 4b) core level spectra show two components. For the N1s region, they are located around 398.5 and 397.1 eV, and for the B1s region around 190.8 and 189.6 eV. This result is very similar to what has recently been reported by Yang *et al*.^53^ for h-BN synthesized on Ni(111) by CVD. According to this report, the existence of epitaxial (and thus tightly bound to the Ni) and non-epitaxial (weakly bound) h-BN islands on the Ni surface is the origin of the high- and low-binding energy components, respectively. A strong correlation between the N1s and B1s binding energies and the interaction between h-BN and the underlying metal template has also been observed for the cases of Ru, Rh, Ir, and Pt^54^,^55^,^56^,^57^. An analogous scenario is likely in our h-BN samples, where different regions of h-BN interact differently with the underlying Ni surface^58^. Although a detailed discussion of this topic is outside the scope of this paper, we can anticipate that h-BN grown on the atomically flat (111) terraces is tightly coupled to the metal surface^59^, whereas a weaker interaction probably takes place at step edge clusters and other disordered regions^60^.

The results obtained from a heterostructure sample exhibit marked differences in the N1s and B1s spectral regions. They are both now composed of a dominant component with binding energies around 398.3 (N1s) and 190.7 eV (B1s). These values are in good agreement with previously published data for h-BN on top of graphene^10^,^11^. The existence of a weak component located at ~399.6 eV in the N1s region is interpreted as being due to N-C bonding^43^,^61^, whereas the B1s contributions at ~188.3 eV and 192 eV can be associated with the existence of elemental and oxidized B^62^,^63^. Although a B/N intensity ratio around 1 was found, as expected for stoichiometric h-BN, a slight excess of surface B might be the reason for these observed contributions^30^. Note that despite not being observed in spectra from the purely h-BN film (probably due to the much higher intensity of the double-shape h-BN related peaks), excess surface B (and associated oxidized B) is also expected for this sample.

The existence of graphene in the heterostructure film is confirmed by measuring the C1s spectral region (Fig. 4c), which is dominated by a component with a binding energy around 284.6 eV associated with the *sp*^*2*^ bonded C atoms within graphene^10^,^11^,^64^. The shoulder observed on the higher binding energy side is due to an additional component located near 285.8 eV, and has been correlated to bonding between C and N atoms^43^,^65^,^66^. The weak component observed at ~288.9 eV can be attributed to a slight CO contamination in the heterostructure films, and has also been observed for pure h-BN films^66^,^67^. The formation of C−B bonds, which should result in components appearing at a lower binding energy in the C1s region (283–283.5 eV), and in the 187–188 eV range for the B1s level^50^,^52^,^62^, were not detected. This suggests that a weak N doping of the graphene film during the initial stages of the h-BN growth is indeed the main reason for the splitting of the G peak observed by UV Raman spectroscopy.

Importantly, the transition from two-component to one-component XPS spectra reveals that the heterostructure formation leads to a more homogeneous interaction of the h-BN film with the underlying template, a result of the continuous few-layer graphene coverage formed at the h-BN/Ni interface via C precipitation. It indicates that our synthesis approach results in the growth of graphene underneath the h-BN, even in regions where the first h-BN atomic layer is strongly bound to the Ni surface. This is different from what has recently been reported for the CVD-based synthesis of graphene under h-BN, in which graphene could only be formed under weakly bound h-BN islands^53^. Hence, in order to get initial insights on the feasibility of such constricted interfacial graphene growth as observed here, it is interesting to consider aspects related to the growth of graphene films by MBE on the same type of Ni/MgO(111) templates. Wofford *et al*.^29^ have shown previously that graphene starts to grow at step edge clusters and then propagates to the terraces to form an extended layer. Sequential growth (from below) of further graphene layers will also initiate at the step edge regions^29^,^31^. In the present case, even though the Ni surface is covered by h-BN, a similar formation mechanism appears to be feasible. This is a result of the expected weaker interfacial bond between h-BN and Ni in regions containing step edge clusters in comparison to the strong interaction present in Ni(111) regions^60^. Interestingly, for graphene this interaction remains strong regardless of the Ni surface region^60^. In this way, the existence of weakly bonded h-BN coverage at step edge clusters should not constitute a barrier for the formation of underlying graphene at this region. However, the formation of graphene at the h-BN covered Ni(111) terraces is somewhat more intriguing, given the similar origin of the strong chemical bonding occurring at h-BN/Ni(111) and graphene/Ni(111) interfaces (a hybridization between Ni *d* states and *π* states of the 2D layer)^68^,^69^,^70^,^71^. In spite of their shared nature, Oshima *et al*.^72^ suggest that stronger interfacial bonding takes place between graphene and Ni. Such a difference becomes significant when considering the two paradigms through which graphene may grow during C precipitation at the h-BN/Ni interface: as thicker graphene/graphite deposits (possibly confined to step edge clusters), or as a continuous film (as is observed experimentally). It is illustrative to differentiate these two modes using a simple energy balance, analogous to traditional thin film growth on an exposed substrate surface (i.e. Volmer-Weber (VW) *vs.* Frank-van der Merwe (FM) growth)^73^. There are four interfacial energies (γ) pertinent to the balance: the bonding strength between h-BN and Ni (γ_h-BN/Ni_), between graphene layers (γ_Gr/Gr_), between graphene and h-BN layers (γ_Gr/hBN_), and between graphene and Ni (γ_Gr/Ni_). Given the similar van der Waals interactions between layers in both graphene and h-BN – and its small absolute magnitude – the strength of the bonding between each material and the Ni surface is likely to dominate. If the energetic driving force to maintain the h-BN/Ni bonding is larger than the graphene/Ni binding energy:


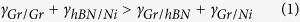


the graphene will preferentially form thicker multilayer deposits, minimizing the total area of h-BN which is decoupled from the Ni surface upon C intercalation (similar to VW growth mode). However, if the graphene/Ni binding energy is larger:


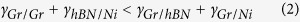


the formation of a continuous graphene film will be more favorable (comparable to FM growth mode). The observation of the continuously detected graphene Raman signal across the sample surface proves that the C precipitated as a continuous graphene film, consistent with the behavior expected from the h-BN/Ni and graphene/Ni interactions^72^. Note that this analysis assumes that thermodynamics rather than kinetics dominating the growth, which is reasonable given the extremely slow cooling rate used during the precipitation process (4 °C/min).

**Figure 5:**
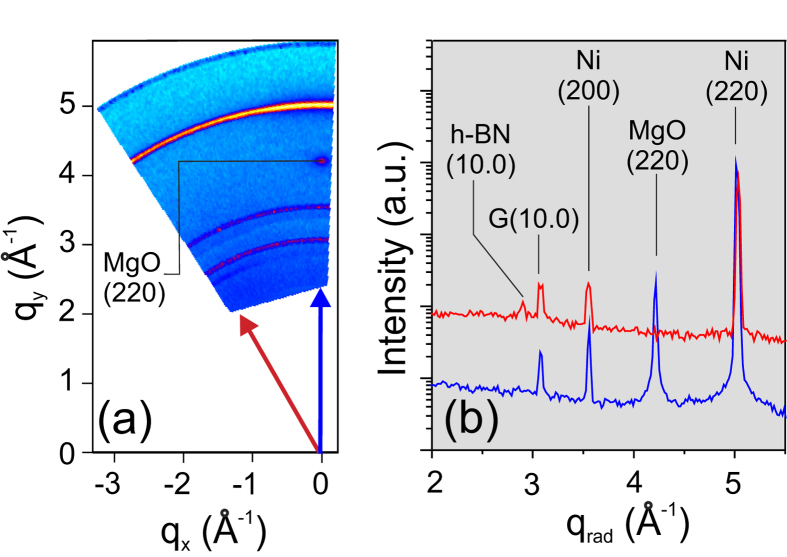
(**a**) GID reciprocal space map, and (**b**) radial scans from a h-BN/graphene heterostack grown on Ni/MgO(111) (see blue and red arrows in (**a**)). The Ni related diffraction in (**a**) forms rings, showing that there is rotational disorder in the Ni film, with a preferential orientation of Ni(220) being parallel to MgO(220). Although difficult to resolve in (**a**), diffraction peaks from both h-BN and graphene are easily observed in (**b**).

Both constituent materials of the h-BN/graphene heterostructures could also be detected via GID. This technique has proven to be very powerful in obtaining precise information about the structural properties of single graphene layers^74^. Figure 5a shows a reciprocal space map of the sample surface, which is dominated by intense diffraction from the larger volume of the Ni film and MgO(111) substrate. The ring-like structure of the Ni-related features indicates a polycrystalline film composed of (111) oriented domains, with a preferential orientation of Ni(220) parallel to MgO(220) visible in the intensity modulation along the ring (see also Fig. S4, Supplementary Information). Examining two radial scans – one along the <110> direction (Fig. 5b, blue) and one rotated azimuthally by 30° (Fig. 5b, red) – allows the comparatively weak h-BN and graphene reflections to be resolved. The diffraction peak at 2.899 Å^−1^ results from the (10.0) reflection of the h-BN lattice, and corresponds to an in-plane lattice spacing of 2.502 Å which is very close to its bulk value^75^. Moreover, the h-BN peak shows a stronger structural correlation with the Ni surface as the arc formed by its (10.0) diffraction is centered on the 30° rotated scan. This is consistent with its growth occurring on the bare metal^69^. The (10.0) reflection of graphene at 3.085 Å^−1^, however, refers to an average real-space in-plane lattice parameter of 2.352 Å, and reveals that the graphene lattice is strongly compressed in about 4% (in comparison to graphite - a = 2.461 Å)^74^. The direct contact between graphene and Ni(111) leads to a compression of the graphene lattice during cooling because of the different thermal expansion coefficients of graphene and Ni^32^. Nevertheless, this effect alone cannot account for the lattice shrinkage measured by GID. Substitutional incorporation of N atoms in the graphene lattice is most likely the dominant effect behind it, since the length of the C-N covalent bond is shorter (1.32 Å) than that of the C-C one (1.42 Å)^76^. Additionally, N incorporation in pyridinic and pyrrolic sites^43^,^61^,^66^, which is also likely to take place, will result in the creation of vacancies in the graphene lattice. This type of point defect is known to contribute to a reduction of the average lattice parameter in graphene^77^. Note that the lattice parameter as well as the general blue shift of the G peak of graphene observed here are very similar to what have been reported by Zafar *et al*^43^. for N-doped graphene with an average N concentration of ~3% prepared by CVD on Cu substrates. Overall, the results obtained by GID corroborate the Raman and XPS findings. As previously mentioned, the preference for N incorporation instead of B is probably associated with the higher reactivity of the N species generated by the plasma source during the growth of the h-BN layers. Finally, the GID data show that the graphene film contains a larger rotational disorder in comparison to the h-BN one (as illustrated by its presence in both radial scans). The extent of rotational disorder in the graphene agrees with previous reports which show that growth on Ni at substrate temperatures above ~650 °C reduces the likelihood of epitaxy^78^.

## Summary

We have demonstrated a novel growth method for the production of layered h-BN/graphene heterostructures based on MBE. The utilization of a Ni film pre-saturated with C as a substrate for h-BN deposition is shown to enable the growth of few-layer graphene at the h-BN/Ni(111) interface by controllably ramping the temperature down to precipitate the C out of the metal. The primary benefit of this technique is that both constituent materials of the heterostructure form in the chemically favorable environment offered by the Ni surface, resulting in continuous graphene and h-BN layers. The top h-BN layer in the stack is strain free, consistent with it being decoupled from the Ni substrate due to the interfacial graphene growth. A careful analysis of the material properties also revealed a significant contraction of the graphene’s crystal lattice due to N-doping. We correlate the N-doping to the possible existence of undissolved C atoms and graphene inclusions present at the Ni surface at the moment when h-BN starts to be grown. Further adjustment of growth parameters shall be implemented in order to mitigate this effect. In terms of thickness homogeneity, it is anticipated that the use of Ni films with smoother surfaces and thus a lower density of step clusters will be beneficial. The h-BN/graphene heterostructure film could be transferred to a SiO_2_/Si substrate without having its structural properties substantially affected, as verified by Raman spectroscopy. In general, the procedure described here offers an alternative route for the scalable production of h-BN/graphene heterostructures which overcomes many of the drawbacks faced by other methods. It also opens a new pathway for the production of other heterosystems composed of graphene in combination with another material, especially in cases where the formation of a homogeneous graphene underlayer (e.g. as a bottom electrode) might be required.

## Methods

For the pure graphene and h-BN films whose Raman results are depicted in Fig. 3a, the following parameters were employed. For graphene: a C flux equivalent to few layer graphene (using an e-beam heated HOPG target, 99 mA, 5 kV, 40 min) was introduced to the Ni surface at 730 °C. A cooling rate of 4 °C per minute is used for C precipitation. For h-BN synthesis, a high-temperature effusion cell operating at ~1850 °C provides the elemental B while reactive N is generated using an RF plasma source working at 0.2 sccm N_2_ flow and 350 W power. The h-BN growth duration is 5 hours. More details for the h-BN growth can be found elsewhere^30^. The growth parameters for h-BN/graphene heterostructures were the same as the growth of individual pure graphene and pure h-BN with the sequence described in the text and summarized in Fig. 1. Several heterostructure samples were prepared with the same conditions and thoroughly characterized by AFM and Raman spectroscopy. From these samples one sample was additionally investigated by XPS and one sample by synchrotron-based GID.

Micro Raman spectroscopy was employed to investigate the structural properties as well as the lateral continuity of the grown material. Excitation wavelengths (λ) in the visible (473 nm) and ultraviolet (244 nm) regions were employed.

AFM was used to examine the surface morphology of the resulting heterostructure films.

XPS was employed to investigate the chemical composition and binding states of the heterostructures. The samples were transferred in air for the XPS measurements. The measurements were performed using a Scienta ESCA 200 spectrometer with a base pressure of 7.5 × 10^−11^ Torr. The chamber is equipped with a monochromatic Al(K alpha) x-ray source providing photons with 1486.6 eV for XPS. The experimental condition was set so that the full width at half maximum (FWHM) of the clean Au 4f7/2 line was 0.65 eV. All spectra were collected at room temperature with a photoelectron takeoff angle of 0° (normal emission) and without sample pre-annealing.

Grazing incidence X-ray diffraction (GID) was used to evaluate the crystalline configuration of the surface layers of the heterostructure. These experiments were performed at beamline BM25B (SpLine) at the European Synchrotron Radiation Facility, Grenoble, France. The X-ray energy used was 20 keV, with the beam at a 0.1° angle of incidence to ensure a high degree of surface sensitivity.

## Additional Information

**How to cite this article**: Wofford, J. M. *et al*. A hybrid MBE-based growth method for large-area synthesis of stacked hexagonal boron nitride/graphene heterostructures. *Sci. Rep.* **7**, 43644; doi: 10.1038/srep43644 (2017).

**Publisher's note:** Springer Nature remains neutral with regard to jurisdictional claims in published maps and institutional affiliations.

## Supplementary Material

Supplementary Information
