# Supplementary material for: A hybrid MBE-based growth method for large-area synthesis of stacked hexagonal boron nitride/graphene heterostructures
Source: Sci Rep. 2017 Feb 27;7:43644. doi: 10.1038/srep43644 (PMC5327397; doi:10.1038/srep43644)
Supplement: Supplementary Information [file srep43644-s1.pdf]

Supplementary Information

A hybrid MBE-based growth method for large-area synthesis of stacked hexagonal boron nitride/graphene heterostructures

Joseph M. Wofford,[1,†] Siamak Nakhaie,[1,†] Thilo Krause,[1] Xianjie Liu,[2] Manfred Ramsteiner,[1] Michael Hanke,[1] Henning Riechert,[1] J. Marcelo J. Lopes[1,*]

[1] Paul-Drude-Institut für Festkörperelektronik, Hausvogteiplatz 5-7, 10117 Berlin, Germany

[2] Department of Physics, Chemistry and Biology, Linköping University, SE-58183 Linköping, Sweden

[†] Contributed equally to this work

[*] Corresponding author: lopes@pdi-berlin.de

Transferring the heterostructures onto SiO2/Si substrates

A standard wet chemical transfer technique based on a method published previously[1] was used to transfer the 1cm × 1cm h-BN/graphene heterostructure film from the growth substrate [Ni/MgO(111)] onto SiO_2/Si substrates. For this, a PMMA solution (PMMA: Alfa Aesar 43982, concentration: 40 mg/ml in chlorobenzene) was spin-coated (3000 rpm, 1 minute) onto the heterostructures. Dilute nitric acid (10%) was used as Ni etchant. The PMMA/h-BN/graphene stack was then rinsed in water, moved onto a SiO_2/Si substrate and baked at 150 °C for 5 min on a hot plate. The PMMA was finally removed using acetone.

Raman spectroscopy on transferred heterostructures:

Raman spectroscopy was used to study the h-BN/graphene heterostructures which were transferred onto SiO_2/Si substrates (Fig. S1 and S2). Similar to as-grown heterostructures, the G and 2D peak of graphene was also observed in all spectra obtained from the transferred heterostructures, re-confirming the presence of a continuous graphene coverage across the heterostructure film. Moreover, a Raman mapping using the 473 nm laser was facilitated, due to the absence of the inhomogeneous Ni background luminescence. An optical micrograph of the transferred h-BN/graphene film corresponding to the region mapped by Raman is presented in Fig. S2a. The regions with higher brightness are related to thickness inhomogeneity in the h-BN and/or in the graphene layers. Raman maps of the D peak intensity over G peak intensity (I_D/I_G) and 2D peak intensity over G peak intensity (I_{2D}/I_G) measured over the exact same area shown in Fig. S2a are presented in Fig S2c. and Fig S2d. Please note that the transfer process may induce additional defects in the structure of the graphene layers, affecting the D peak intensity.

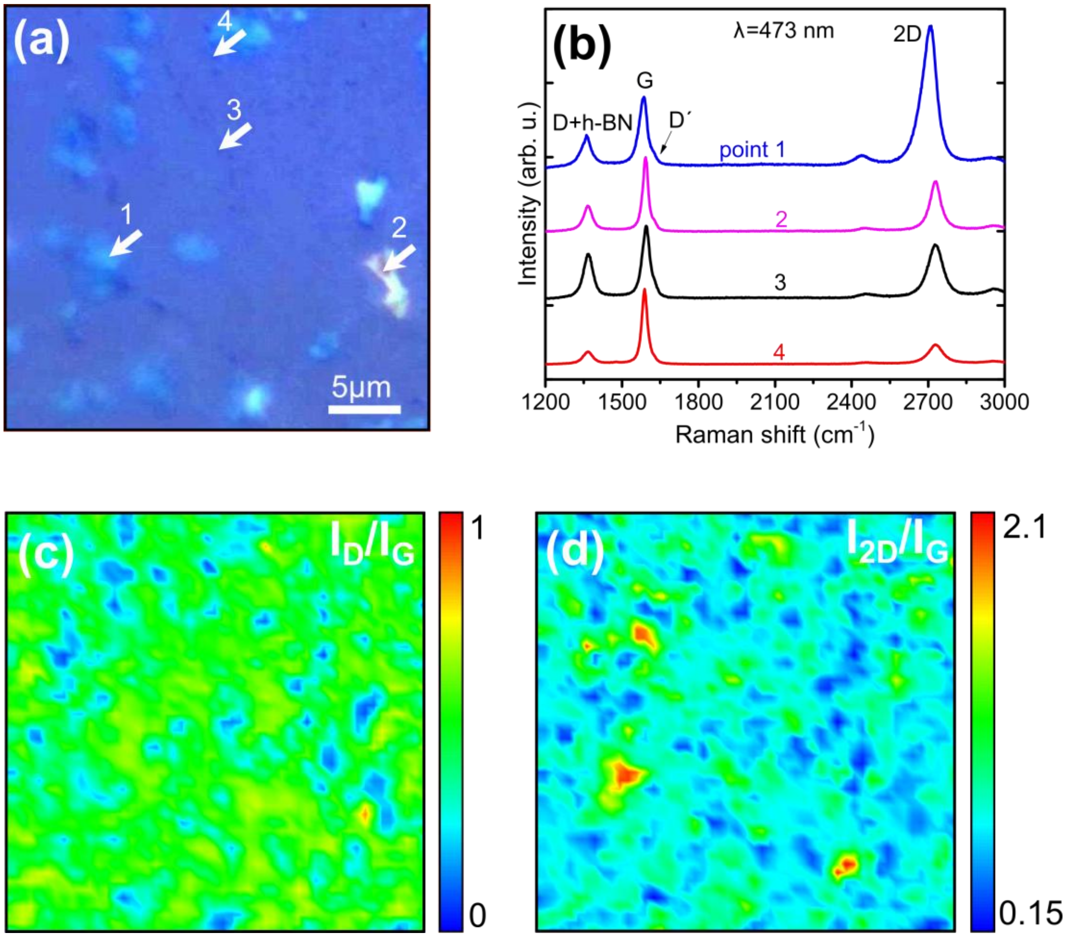

Fig. S1. (a) Optical micrograph (30μm × 30μm) of the h-BN/graphene heterostructure transferred onto SiO_2/Si wafer corresponding to the surface area probed by Raman spectroscopy. The brightness and contrast of the image is manually enhanced for a better demonstration. **(b)** Raman spectra of the points shown by arrows in **(a)** using a 473 nm laser. **(c)** and **(d)** show the Raman maps of the D peak intensity over G peak intensity (I_D/I_G) and 2D peak intensity over G peak intensity (I_{2D}/I_G) measured over the exact same area presented in **(a)**, respectively. Please note that the intensity measured for the D peak also

includes a small contribution from the h-BN peak (at ~1364 cm^{-1}).

UV Raman spectroscopy allows us to confirm the presence of h-BN also in the transferred h-BN/graphene heterostructure. The h-BN's Raman peak at ~1364 cm^{-1} is always observed in UV Raman measurements obtained from the transferred heterostructures. Figure S2 shows the position of h-BN peak over a 30 μm × 30 μm mapped area. Please note that the D peak of graphene is shifted to higher wavenumbers (~1485 cm^{-1}) and quenched when the UV laser (244 nm) is used, as discussed in the main manuscript text.

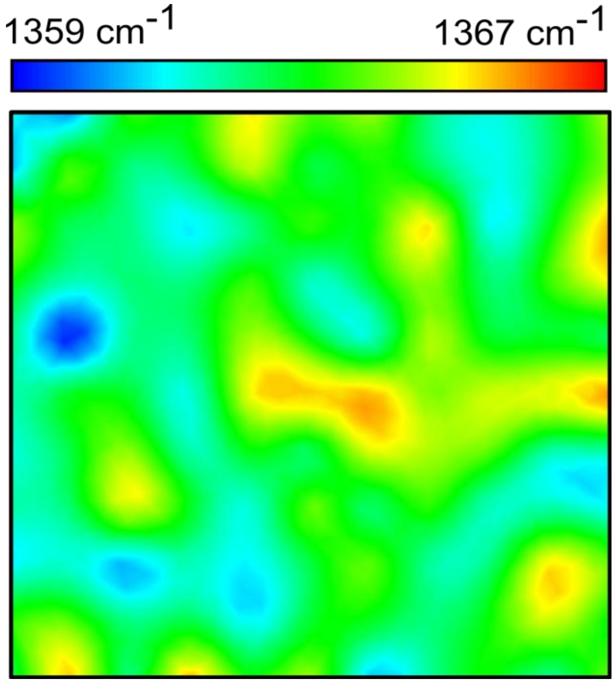

Fig. S2. Raman map of h-BN's peak position obtained from the transferred heterostructure using the 244 nm laser (same sample used to obtain the maps presented in Fig S1).

O_2-plasma treatment of the transferred samples:

In order to confirm the stacking order of h-BN/graphene heterostructure, O_2-plasma treatments were performed. For this, half of a transferred 1cm × 1cm h-BN/graphene heterostructure was protected with a solid mask and half was left uncovered. The sample was then exposed to O_2-plasma etching (100W, 2 minutes, O_2 pressure: 0.5 mbar), which would remove any surface graphene from the uncovered area.[2,3] The sample was then checked with Raman spectroscopy. The G peak of graphene is observed in both protected and uncovered areas, showing that the Raman signal originates from graphene formed under the h-BN layers, as expected.

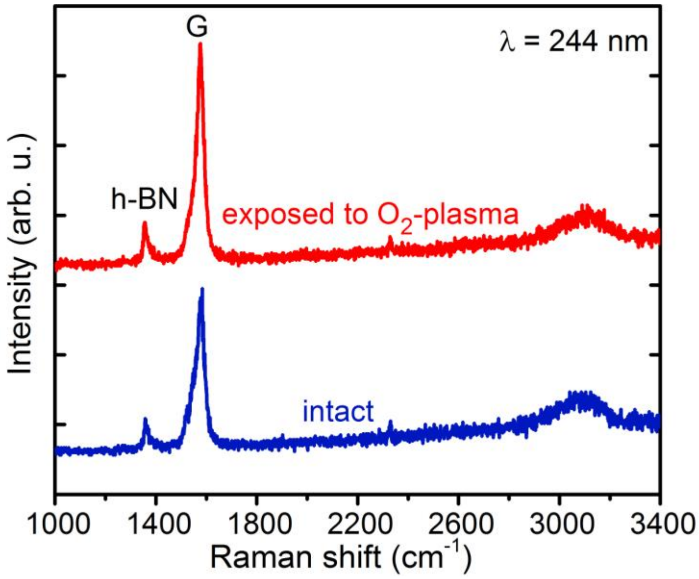

Fig. S3. Raman spectra from a transferred h-BN/graphene heterostructure using the UV (244 nm) laser. The red spectrum is acquired from a region which was exposed to O_2-plasma. The blue spectrum is acquired from a region which was protected from the plasma.

GID Ni film characterization

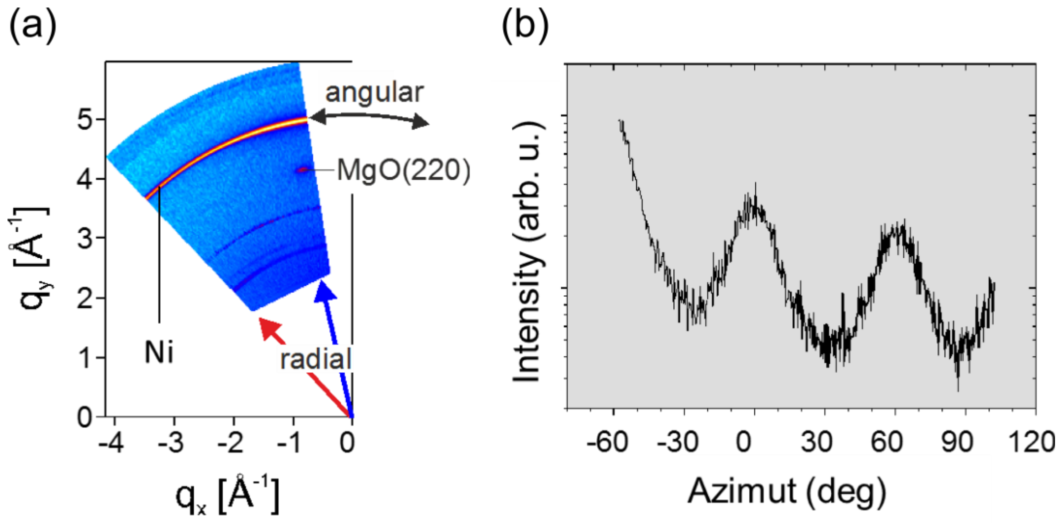

Fig. S4. (a) GID reciprocal space map (similar to the one shown in paper). **(b)** Intensity profile obtained from the angular scan along the ring like Ni contribution in (a). There is a preferential alignment of Ni[110] || MgO[110], however Ni has a textured character.

References:

1. Suk, J. W. *et al.* Transfer of CVD-grown monolayer graphene onto arbitrary substrates. *ACS Nano* **5,** 6916–6924 (2011).

2. Schumann, T. *et al.* Anisotropic quantum Hall effect in epitaxial graphene on stepped SiC surfaces *Phys. Rev. B* **85**, 235402 (2012).

3. Oliveira, M. H. *et al.* Mono- and few-layer nanocrystalline graphene grown on Al_2O_3(0001) by molecular beam epitaxy. *Carbon* **56,** 339–350 (2013).
